# Butyrylcholinesterase–Protein Interactions in Human Serum

**DOI:** 10.3390/ijms221910662

**Published:** 2021-10-01

**Authors:** Jacek Jasiecki, Anna Szczoczarz, Dominik Cysewski, Krzysztof Lewandowski, Piotr Skowron, Krzysztof Waleron, Bartosz Wasąg

**Affiliations:** 1 Department of Pharmaceutical Microbiology, Faculty of Pharmacy, Medical University of Gdańsk, 80-416 Gdańsk, Poland; krzysztof.waleron@gumed.edu.pl; 2 Department of Pharmaceutical Pathophysiology, Faculty of Pharmacy, Medical University of Gdańsk, 80-416 Gdańsk, Poland; anna.szczoczarz@gumed.edu.pl; 3 Mass Spectrometry Laboratory Institute of Biochemistry and Biophysics, Polish Academy of Sciences, 02-106 Warsaw, Poland; dominikcysewski@gmail.com; 4 Department of Laboratory Medicine, Medical University of Gdańsk, 80-952 Gdańsk, Poland; krzysztof.lewandowski@gumed.edu.pl; 5 Department of Molecular Biotechnology, Faculty of Chemistry, University of Gdańsk, Wita Stwosza 63, 80-308 Gdańsk, Poland; piotr.skowron@ug.edu.pl; 6 Department of Biology and Medical Genetics, Medical University of Gdańsk, 80-210 Gdańsk, Poland; bwasag@gumed.edu.pl; 7 Laboratory of Clinical Genetics, University Clinical Centre, 80-952 Gdańsk, Poland

**Keywords:** BChE, butyrylcholinesterase, pseudocholinesterase, high-density lipoprotein (HDL), protein interactions, ApoA-I, protein interactions

## Abstract

Measuring various biochemical and cellular components in the blood is a routine procedure in clinical practice. Human serum contains hundreds of diverse proteins secreted from all cells and tissues in healthy and diseased states. Moreover, some serum proteins have specific strong interactions with other blood components, but most interactions are probably weak and transient. One of the serum proteins is butyrylcholinesterase (BChE), an enzyme existing mainly as a glycosylated soluble tetramer that plays an important role in the metabolism of many drugs. Our results suggest that BChE interacts with plasma proteins and forms much larger complexes than predicted from the molecular weight of the BChE tetramer. To investigate and isolate such complexes, we developed a two-step strategy to find specific protein–protein interactions by combining native size-exclusion chromatography (SEC) with affinity chromatography with the resin that specifically binds BChE. Second, to confirm protein complexes′ specificity, we fractionated blood serum proteins by density gradient ultracentrifugation followed by co-immunoprecipitation with anti-BChE monoclonal antibodies. The proteins coisolated in complexes with BChE were identified by mass spectroscopy. These binding studies revealed that BChE interacts with a number of proteins in the human serum. Some of these interactions seem to be more stable than transient. BChE copurification with ApoA-I and the density of some fractions containing BChE corresponding to high-density lipoprotein cholesterol (HDL) during ultracentrifugation suggest its interactions with HDL. Moreover, we observed lower BChE plasma activity in individuals with severely reduced HDL levels (≤20 mg/dL). The presented two-step methodology for determination of the BChE interactions can facilitate further analysis of such complexes, especially from the brain tissue, where BChE could be involved in the pathogenesis and progression of AD.

## 1. Introduction

Blood serum is a fluid that contains 60–80 mg of protein/mL in addition to various small molecules, including salts, lipids, amino acids, and sugars. It is estimated that up to 10,000 proteins may be present in serum, ranging in concentration over at least 9 orders of magnitude. Blood serum proteins serve various functions, including transport of lipids, hormones, and vitamins. They are responsible for maintaining acid–base balance, oncotic pressure, plasma viscosity, and the functioning of the immune system. The major protein constituents of serum include albumin, immunoglobulins, transferrin, haptoglobin, and lipoproteins [1,2]. In addition to these major constituents, proteins can also be released into the serum from cells and tissues as a result of disease processes, apoptosis, necrosis, and hemolysis. The presence of these components in blood makes it possible to use targeted proteomics to detect biomarkers of disease states [3,4].

Out of the several hundred different blood serum proteins, only a few are determined by laboratory diagnostics. One of the serum proteins, butyrylcholinesterase (BChE, EC 3.1.1.8), also known as plasma cholinesterase or pseudocholinesterase, is a serine hydrolase present in almost all mammalian tissues with the highest levels detected in plasma and liver [5,6]. BChE hydrolyzes acetylcholine and all chemicals containing ester bonds, of sufficient size to fit into the active site pocket, including anesthetics such as succinylcholine and procaine and drugs such as cocaine and heroin [7,8,9,10,11]. Moreover, BChE in the plasma serves as the first line of defense against toxic compounds reaching the bloodstream that might inhibit acetylcholinesterase (AChE) activity. BChE sequesters (irreversibly binds) acetylcholinesterase inhibitors acting at the neuromuscular junction such as organophosphates (OPs), warfare nerve agents, and pesticides, thus protecting the nervous system before the poisons reach their target, i.e., synaptic AChE, and exert effects [12]. From a clinical point of view, BChE is a perfect indicator of pesticide poisoning and nerve agent exposure because lowered ChE plasma activity is the first sign of the toxic effect of these compounds that is easy and convenient to monitor [13,14]. BChE is also a target enzyme for cholinesterase inhibitors, which increase the availability of neurotransmitters at synapses in the brain and are used clinically as the mainstay of Alzheimer’s therapy [15]. Nevertheless, the exact physiological function of BChE remains intangible, but research over the past two decades has revealed new roles for BChE. It has been shown that BChE hydrolyzes the hunger hormone ghrelin, thus reducing obesity, as well as aggression and social stress in mice [16,17,18]. BChE is found in glia and white matter in the brain and is also associated with neurons, particularly in the hippocampus, amygdala, and thalamus [19,20]. BChE was also found in amyloid plaques and neurofibrillary tangles (NFTs), which suggests that the protein may be involved in the pathogenesis of AD [21,22]. BChE is present in human plasma with a concentration of 2–5 mg/L mainly as glycosylated soluble tetramers with a molecular weight of 340 kDa [23,24,25]. Four identical subunits assemble into a tetramer by the BChE tetramerization (WAT) domain at the C-terminus. In small portions, BChE also occurs as dimers and monomers. The BChE tetramer is organized as a nonplanar dimer of dimers arranged around a central helical oligopeptide with a proline-rich attachment domain (PRAD) that is shielded by the four catalytic domains, which may stabilize the enzyme [26,27]. Most, ~70%, of the proline-rich peptides are derived from lamellipodin, and the rest are derived from other proteins [28]. New translated BChE molecules are released into the plasma as monomers, which then associate polyproline-rich peptides with forming the functional tetramer, increasing its stability and half-life. BChE also occurs in forms bound to cellular membranes by the proline-rich membrane anchor (PRiMA). Furthermore, there are more complex forms of BChE that consist of two, three, or four tetramers, A4, A8, and A12, that are attached to membranes by a proline-rich attachment domain of collagen Q anchor [29,30,31]. It has also been reported that ChE can form hybrids with both AChE and BChE catalytic subunits [32,33].

Interestingly, our previous work showed that the serum dilution factor influences BChE activity [34]. The more diluted the serum sample was in phosphate buffer, the higher BChE activity was observed. That study has indicated that some factors of such a multicomponent clinical material like a serum can influence kinetic parameters of the BChE. The results indicated that the putative inhibitor probably is larger than 3 kDa, so it could be one or more proteins or other large molecules. Since the serum is protein-abundant, it is not surprising that such interactions can be formed. This leads to the question of whether BChE interacts with specific proteins and forms much larger complexes than predicted from the molecular weight of the tetramer. To investigate and isolate such complexes, we employed two independent two-step strategies based on the combined separation method with affinity chromatography using resins that bind BChE. Using these two approaches, we aimed to ensure that detected interactions are confirmed and are the most probable.

## 2. Results

### 2.1. Isolation of BChE–Protein Complexes by Size-Exclusion Chromatography (SEC) of Serum Proteins 

**Figure 1:**
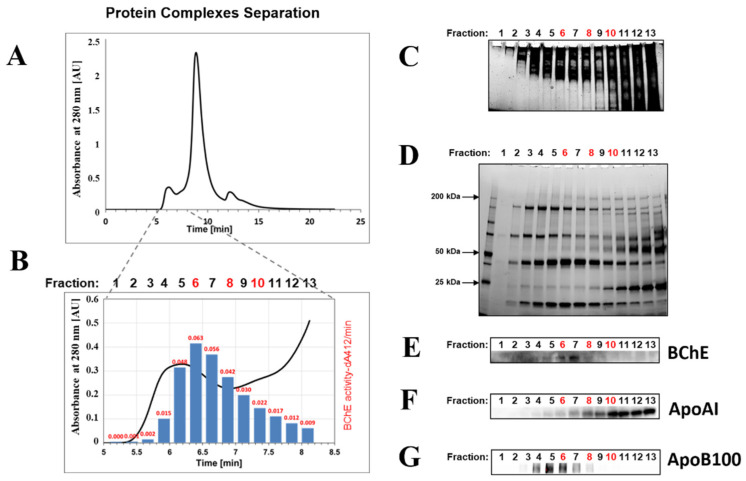
Isolation of the BChE–protein complexes by size-exclusion chromatography (SEC) of serum proteins. (**A**) Chromatogram of human serum proteins separated on the TSK gel 3000 SWXL (Tosoh, Tokyo, Japan). Protein elution was monitored at 280 nm. The first 13 fractions of 200 µL were collected every 12 s in separate tubes. (**B**) Determination of the BChE activity in the fractions by Ellman’s assay in the collected fractions. The BChE activity is shown as dA412/min in red numbers above corresponding bars. dA412/min stands for change in absorbance at 412 nm per minute. (**C**) Analysis of the protein complexes in the 13 fractions by native polyacrylamide gel electrophoresis on Mini-PROTEAN 4–15% TGX Stain-Free Gels (Bio-Rad, Hercules, CA, USA). Ten microliters of each fraction was separated under native conditions in TB buffer at 4 °C. (**D**) SDS-PAGE analysis of the protein complexes in the 13 fractions. Ten microliters of each fraction sample was separated on Mini-PROTEAN 4–15% precast TGX Stain-Free gels (Bio-Rad, Hercules, CA, USA). M—protein marker; 1–13—10 µL of fractions. (**E**) Western blot analysis of 13 fractions using mouse monoclonal anti-BChE antibody (D-5): sc-377403 Santa Cruz Biotechnology (Santa Cruz, CA, USA) (1/200 dilution). (**F**) Western blot analysis of the fractions using rabbit monoclonal anti-ApoA-I antibody (ab52945) (Abcam, Cambridge, UK) (1/2000 dilution). (**G**) Western blot analysis of the fractions using rabbit monoclonal anti-ApoB antibody (ab139401) (Abcam, Cambridge, UK) (1/2000 dilution).

Our published work suggests that BChE can form larger complexes with other proteins [34]. To investigate the specificity of BChE complexes, we first separated serum by size-exclusion chromatography (SEC) (Figure 1A,B). Fractions from the column were collected, and the BChE activity was assessed in each sample by Ellman’s method. As expected, BChE activity was observed in several first fractions from the SEC column. The fractions were analyzed by protein gel electrophoresis in native and denaturing conditions to visualize protein complexes (Figure 1C,D). The sizes of protein complexes observed in native gel electrophoresis were consistent with retention time from the column, and the largest complexes were eluted first. Western blot analysis with anti-BChE antibodies confirmed the presence of BChE in several protein complexes of different sizes, which is consistent with observed enzyme activity (Figure 1B,E). Since serum consists of large portions of lipoproteins, markers of such molecules were searched for in the fractionated complexes. Western blot analysis revealed ApoA-I and ApoB100, which are protein markers of HDL and LDL, respectively. However, the low resolution of the SEC column cannot effectively separate lipoproteins, so the fractions containing LDL and HDL were overlapped (fractions 5–8 in Figure 1F,G).

### 2.2. Binding of BChE–Protein Complexes by Affinity Chromatography on Hupresin AC Sepharose

**Figure 2:**
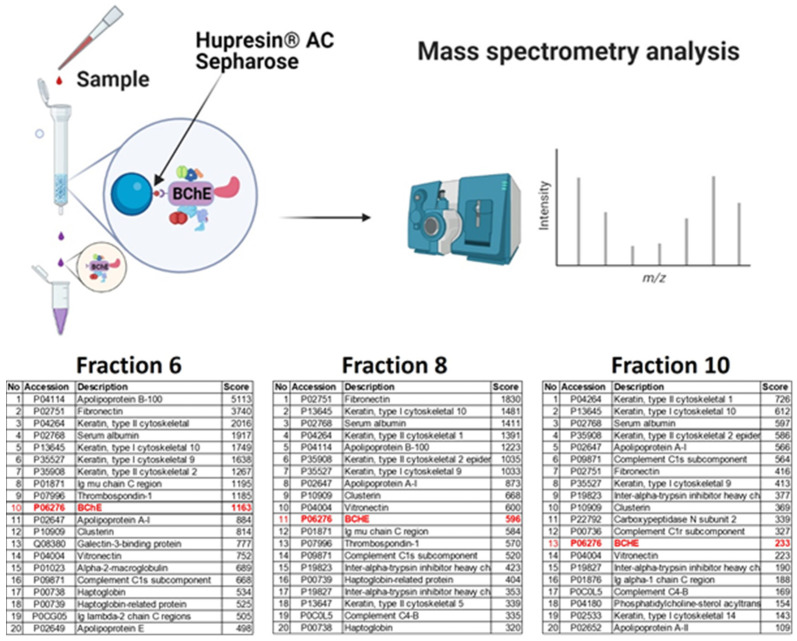
Scheme of the isolation of the BChE–protein complexes by affinity chromatography on Hupresin AC Sepharose. Proteins from fractions 6, 8, and 10 were applied on Hupresin AC Sepharose (Chemforase, Mont-Saint-Aignan, France). The eluted proteins in complexes were identified by mass spectrometry. The tables list the first 20 most abundant proteins identified in the complexes, sorted according to the score number. For each identified protein, Mascot software calculates a score. This number reflects the combined scores of all observed mass spectra that can be matched to amino acid sequences within that protein. The score in the table is a qualitative measure, not quantitative. The higher the score, the more reliable the identification. Figure 2 was created with BioRender.com (access on 15 March 2021).

We next sought to test the hypothesis that the BChE interacts with other proteins in serum. To do so, we pulled down BChE–protein complexes separated on the column by affinity chromatography. Complexes from fractions 6, 8, and 10 from the SEC column were purified on Hupresin AC Sepharose (Chemforase, Mont-Saint-Aignan, France) and analyzed by mass spectrometry. Detected apolipoproteins in separated fractions suggest that the BChE protein can interact with HDL and/or LDL (tables in Figure 2). Besides confirmation of the presence of HDL and LDL lipoproteins, mass spectrometry analysis revealed the presence of many other proteins that are listed in the tables in Figure 2. To study weak interactions, limited steps of washing were used in the procedure of purification. Nonbound proteins were washed out, which was monitored by Bradford protein assays. The washing step was not performed according to the manufacturer’s instructions of standard BChE purification procedure using Hupresin [35]. However, it was limited to minimal volume of washing buffer (using twice 200 µL of washing buffer) required only to wash out most of nonbound proteins from the resin. As expected, BChE in serum can interact with various proteins; some of these interactions are weak and transient, and others are more specific, but some of them are probably also nonspecific. Proteins such as ApoA-I, vitronectin, and clusterin were detected in all pull-down studies from three fractions.

### 2.3. Isolation of BChE–Protein Complexes by Fractionation of Blood Serum Proteins Using Density Gradient Ultracentrifugation

**Figure 3:**
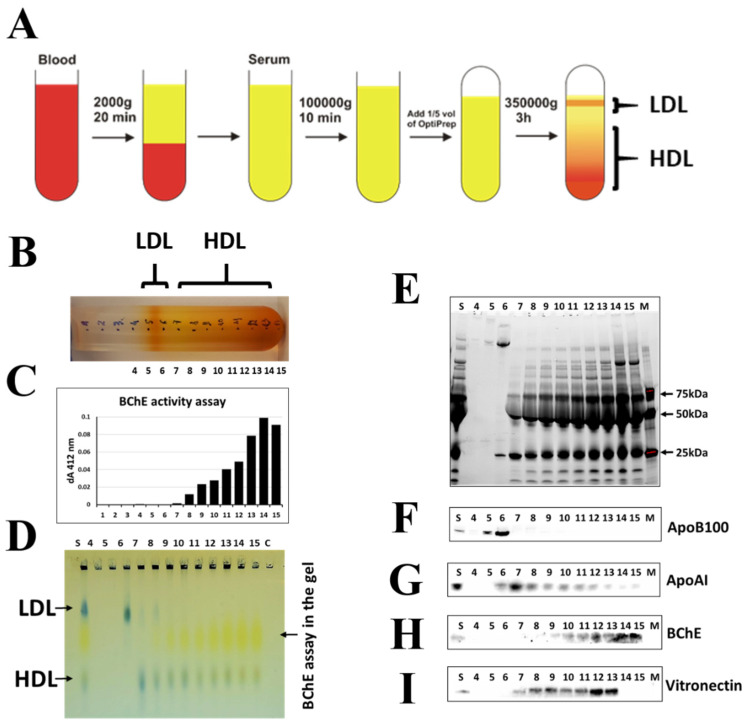
Fractionation of BChE–protein complexes by density iodixanol gradient ultracentrifugation (OptiPrep). (**A**) The separation procedure is summarized in the flow chart. (**B**) Picture of the centrifuged tube taken after separation. Positions of fractions are marked on the tube. (**C**) BChE activity of 10 uL of each fraction was estimated by Ellman’s assay as it has been described in Figure 1B. The BChE activity is shown as dA412/min. (**D**) Sudan black stained agarose gel electrophoresis profile of separated fractions after electrophoresis. Ellman’s reaction was developed in 100 mM PB buffer (pH 7.4) with a final concentration of 0.5 mM DTNB and 5 mM BTC. S—1 µL of serum; 4–15—1 µL of fractions. (**E**) SDS-PAGE; 1 µL of the fraction samples were separated on Mini-PROTEAN 4–15% precast TGX Stain-Free gels (Bio-Rad, Hercules, CA, USA). SDS-PAGE and Western blotting were performed according to the method described in Figure 1D. S—1 µL of serum; M—protein marker; 4–15—1 µL of fractions. (**F**) Western blot analysis of the fractions using rabbit monoclonal anti-ApoB antibody (ab139401) (Abcam, Cambridge, UK) (1/2000 dilution). (**G**) Western blot analysis of the fractions using rabbit monoclonal anti-ApoA-I antibody (ab52945) (Abcam, Cambridge, UK) (1/2000 dilution). (**H**) Western blot analysis of the fractions using mouse monoclonal anti-BChE antibody (D-5): sc-377403 Santa Cruz Biotechnology (Santa Cruz, CA, USA)(1/200 dilution). (**I**) Western blot analysis of the fractions using rabbit monoclonal anti-vitronectin antibody (ab46808) (Abcam, Cambridge, UK) (1/2000 dilution).

In order to confirm the specificity of interactions between BChE and proteins, density gradient ultracentrifugation in iodixanol (OptiPrep) gradient was used to separate protein complexes and assess BChE interactions with lipoproteins in human serum. This method fractionated the serum into 15 fractions, including a clearly visible LDL-containing zone (Figure 3A,B). However, BChE was not detectable in the low-density fraction containing LDL. BChE activity was detected by Ellman’s assay and Western blotting. Most of the serum BChE was present in the denser HDL fractions numbered 8–15 (Figure 3B,C). These fractions contained most of the ApoA-I, a protein marker of HDL. BChE activity and the presence of LDL and HDL were also detected by agarose gel electrophoresis of the collected fractions using Ellman’s assay and Sudan black staining, respectively (Figure 3D). Subsequently, the presence of the protein markers LDL and HDL, BChE, and vitronectin in the collected fractions was confirmed by Western blotting (Figure 3E–I).

### 2.4. Isolation of Protein Complexes by Co-Immunoprecipitation and Identification by Mass Spectrometry

**Figure 4:**
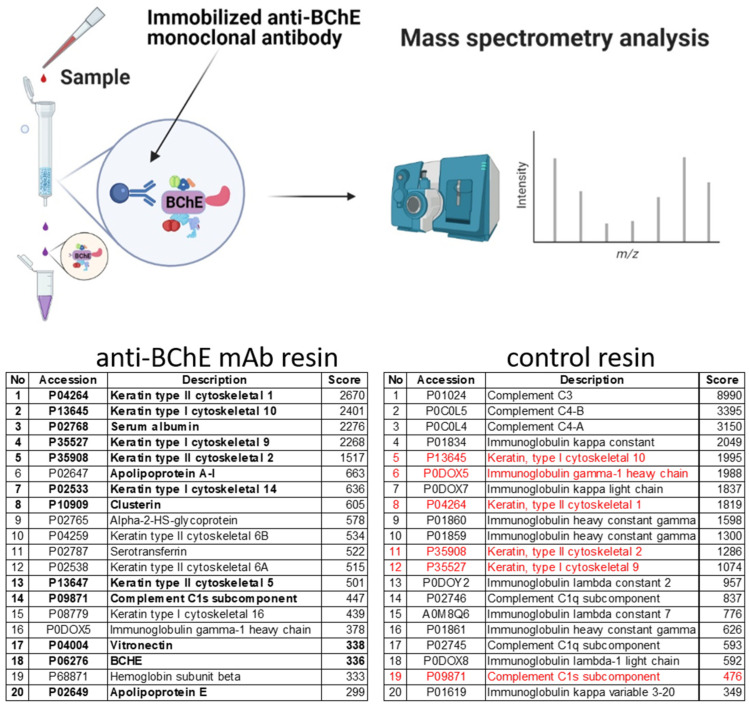
Scheme of the isolation of protein complexes by co-immunoprecipitation, which is a common approach to study protein–protein interactions that uses an antibody to immunoprecipitate the antigen (bait protein) and co-immunoprecipitate any interacting proteins (prey proteins). Isolation of native protein complexes from fraction 12 was performed using immobilized monoclonal antibodies on agarose support according to the instruction of the Thermo Scientific Pierce Co-Immunoprecipitation (Co-IP) Kit (Thermo Fisher Scientific, Waltham, MA, USA). The proteins in complexes were eluted and identified by mass spectrometry. The table lists the first 20 most abundant proteins identified in complexes, sorted according to the score number from the Mascot server. The score in the table is a qualitative measure, not quantitative. The higher the score, the more reliable the identification. The proteins that have also been pulled down using Hupresin AC Sepharose are listed in the table in Figure 2 in bold text. The control resin table lists proteins nonspecifically interacting with the control quenched antibody coupling resin. The proteins identified in both tables (with anti-BChE antibody and in control) are marked in red. Keratin and the immune system proteins seem to bind nonspecifically to the resins in the above pull-down experiments. Figure 4 was created with BioRender.com (access on 15 March 2021).

To verify the hypothesis that BChE can interact with other specific proteins in the serum, we examined whether immunoprecipitation studies could confirm the composition of a potential complex formed between the BChE and HDL proteins or others. Isolation of native protein complexes from fraction 12 from density gradient ultracentrifugation experiment (see above) was performed using immobilized monoclonal mouse anti-BChE monoclonal antibodies on agarose support. As expected, specific antibodies against BChE also pulled down other proteins, including ApoA-I, clusterin, and vitronectin (table in Figure 4). The negative quenched resin control was created by adding 200 μL of quenching buffer to the antibody coupling resin instead of the anti-BChE antibody. The control resin was used in the same way for binding proteins from fraction 12 as in the experiment with the specific antibodies. The eluted proteins were identified by mass spectrometry. In all pull-down experiments, some false-postive results appear caused by nonspecific interactions with the resin matrix. Taken together, the above two pull-down studies revealed that probably all detected keratin and the immune system proteins bind nonspecifically (Figure 4, control resin table, protein list marked in red). On the other hand, the other proteins, i.e., ApoA-I, clusterin, vitronectin, and serum albumin, seem to bind more specifically to BChE protein.

### 2.5. Measurement of BChE Activity in Subjects with Various HDL Levels

**Figure 5:**
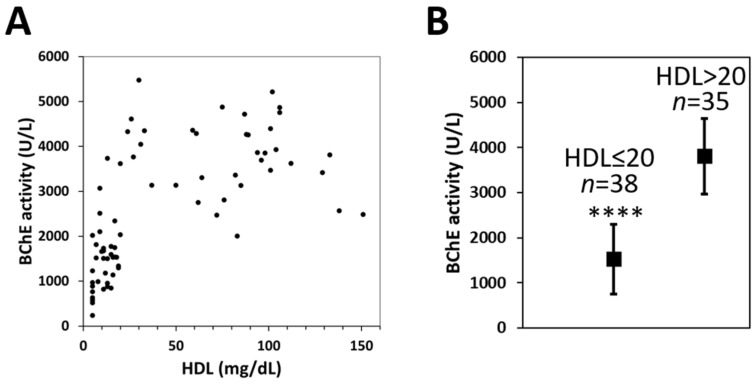
Interrelationship of BChE activity and serum HDL level. (**A**) Each black dot represents the enzyme activity of a single individual. BChE activity was measured by Ellman’s assay as described previously [34]. (**B**) Subjects were divided into 2 groups according to HDL levels. One group (*n* = 38) with HDL level ≤20 mg/dL, the second group (*n* = 35) with HDL level >20 mg/dL; mean values with error bars representing standard deviation (SD), four asterisks (****) represent *p*-value < 0.0001 calculated using the *t*-test.

Since the BChE seems to be bound among others to the main component of HDL, ApoA-I, we tested if HDL concentration reflects BChE level in serum. Therefore, we assessed BChE activity in serum samples from 73 individuals with varying HDL levels. Serum samples with a broad range of HDL levels were obtained from a collection of the Central Clinical Laboratory of the Medical University of Gdansk. We found a visible interdependence in patients (*n* = 38) with low serum HDL levels (≤20 mg/dL) and low BChE activities (below 2000 U/L in 30 patients). On the other hand, at higher HDL levels, the higher but broad range of BChE serum activity (above 2000 U/L) was observed (Figure 5A,B).

## 3. Discussion

The above protein binding studies reveal that BChE can interact with various proteins in human serum. This is not surprising since the serum is a viscous protein-rich medium, but the most surprising is the specificity of the interactions. Based on our previous results showing that the observed serum enzyme activity is dependent on dilution factor [34] and posted hypothesis that the protein interactions are responsible for such a phenomenon, we started deeper studies in this field. First, we observe that BChE forms complexes much larger than the molecular mass of the tetramer, 340 kDa. The density gradient centrifugation and size-exclusion chromatography show that the BChE in the fractions has different large molecular weights rather than one. It suggests that BChE can exist in many serum protein complexes or forms. These two methods of biomolecule separation are based on slightly different rules, so differences in HDL and LDL fractionations were observed (Figure 1F,G and Figure 3F,G). Secondly, specific binding by monoclonal antibody or by Hupresin have revealed that ApoA-I is abundantly present in such complexes, so we are leaning towards the theory that BChE can interact with different forms of HDL, probably via other protein linkers. Although BChE was detected in HDL fractions almost 40 years ago, no more detailed studies of the nature of these interactions were carried out [36]. Limited washing steps during purification on Hupresin may increase the risk of copurification of nonspecifically bound proteins. On the other hand, it allows the detection of weak and transient protein interactions. This approach, however, requires additional experiments to confirm the existence of such interactions. To do this, a similar pull-down study was performed, but in this experiment, monoclonal antibodies were used as an affinity ligand.

ApoA-I is the most abundant protein of HDLs (70% of HDL protein content). It is crucial for the structural and functional integrity of the lipoprotein and is involved in the reverse transport of cholesterol from tissues to the liver and inflammatory and immune response regulation. HDL is a small, dense, protein-rich lipoprotein spherical structure with a diameter of 7.2–13 nm and a density of 1.063–1.21 g/mL (for a review see [37]). An inner hydrophobic core of HDL particles consists of triglycerides (TGs) and cholesteryl esters (CEs), while phospholipids (PLs); sphingomyelins (SMs); unesterified free cholesterol (FC); and apolipoproteins (APOs) including ApoA-I, ApoE, ApoD, and ApoJ (CLU) form the outer the hydrophilic shell. Many other proteins and enzymes such as enzyme lecithin cholesteryl acyltransferase (LCAT), platelet-activating factor acetyl hydrolase (PAF-AH), and paraoxonase 1 (PON1) [38,39] which isalso present in HDL through direct binding of its N-terminal regions to the HDL phospholipids [40]. More than 80 proteins and 150 lipids have been shown to be associated with HDL particles, but each lipoprotein particle probably carries different complements of protein and lipid components.

In the maturation process from nascent small discoidal HDL (preβ-1 HDL) to larger spherical HDL, these proteins and lipids create weaker or stronger, stable or transient interactions with various other molecules, including receptors, enzymes, transporters, serum proteins miRNAs, hormones, and vitamins and metabolites. However, there are no reports of BChE interactions with HDL, suggesting that these interactions are not permanent and strong. The biogenesis of HDL occurs in the liver and intestine, where nascent HDL particles (pre-β-HDL) formed from ApoA-I in cooperation with ABCA1 transporter uptake cholesterol from different cell types. Lipid-rich (mature) HDL particles exchange lipids with chylomicrons, LDL, and VLDL through cholesteryl ester transfer protein (CETP) and with macrophages via ABCG1. Finally, HDL lipids are transferred to the liver via receptors scavenger receptor (SR)-B1 and LDLR after the removal of cholesteryl ester, and the smaller ApoA-I-containing HDL particle dissociates and is recycled. HDL is a highly heterogeneous lipoprotein family consisting of several subclasses differing in size, density, shape, protein, and lipid composition. Differences in HDL subclasses can be observed by using isopycnic density gradient ultracentrifugation with KBr [41] or density gradient medium, iodixanol, commercially available as OptiPrep [42].

We performed separation of the serum according to OptiPrep instructions, and we obtained LDL and broader HDL fractions. While LDL was localized as a visible orange band in two fractions (fractions 5 and 6) at the top of the centrifuge tube, HDL seemed to be widespread in nine fractions (Figure 3B). The distribution of LDL and HDL was been confirmed by Sudan black staining and the detection of specific lipoproteins, respectively ApoB100 and ApoA-I, by Western blotting (Figure 3D,F,G). Observed BChE activity appears to be density gradient-dependent. The denser the fraction is, the more enzyme is present. It turns out that more BChE is colocalized in denser fractions containing HDL3. The specificity of interactions between HDL-associated proteins was also confirmed by two independent pull-down assays using Hupresin and monoclonal antibodies. The results from both assays are congruent in the case of some proteins, i.e., ApoA-I, clusterin, and vitronectin. Surprisingly, all of these proteins have been reported to have interacted with HDL, so we suppose that BChE can weakly interact with HDL rather indirectly by other proteins.

Clusterin (CLU) or ApoJ is a multifunctional glycoprotein linked with AD. Mutations in the CLU gene have been established as the third most predominant genetic risk factor for AD in several genome-wide association studies. Many functions have been ascribed to clusterin, such as molecular chaperone and protein quality control in the extracellular space with the ability to bind Aβ peptides, inhibiting the formation of amyloid fibrils. CLU has been reported to block LDL oxidation and plays additional roles in lipid transport, complement inhibition, regulation of inflammation, appetite regulation, apoptosis, and cell differentiation (for a review, see [43]). Vitronectin, the adhesive glycoprotein, is another HDL-associated protein involved in complement regulation and promotes cells adhesion and spreading [44].

BChE seems to bind to ApoA-I, so we tested if HDL concentration reflects BChE level in the serum. We observed very low BChE activity in 30 patients with extremely low serum HDL levels below 2000 U/L (Figure 5A,B). This suggests that the HDL biosynthesis and secretion process may be engaged partly in BChE secretion, probably by recycled transport. It has been reported that ApoA-I binds to ABCA1 and is internalized into early endosomes that can be either delivered to late endocytic compartments or recycled back to the plasma. Lipidated ApoA-I in late endocytic vesicles is recycled back to the cell surface and released as a nascent HDL particle. In this way, using ABCA1, a pool of lipid-poor ApoA-I is transported from the cell surface to late endocytic vesicles and returns to the cell surface in a lipid-rich state [45,46]. We suppose that some portion of newly synthesized BChE protein can also be exported to the plasma, transported on late endocytic vesicles in contact with ApoA-I. This hypothesis can explain why some portion of the BChE can be detected with N-terminal extended end in human serum in subjects with additional mutations in the *BCHE* gene [47]. Most of the newly translated BChE molecules are probably secreted via conventional transport through the endoplasmic reticulum to the Golgi and then to the plasma membrane, where they are released into the extracellular space. However, some portions of BChE can also be secreted by recycled transport using ABCA1 or unconventional protein secretion (UPS) mechanisms [48].

Our results show that some BChE tetramers exist in the serum as larger complexes in interactions with other proteins. Such interactions can increase the half-life of the enzyme in the bloodstream. The multicomponent enzymatic complex with BChE can also facilitate reaction performance in blood. Similarly, PON1 serves as an antioxidant component of HDL and is responsible for its antioxidative feature. PON1 has been shown to help HDL protect against the development of atherosclerosis by preventing the accumulation of lipoperoxides in LDL, inactivate oxidized lipids, enhance cholesterol efflux from macrophages, and stimulate HDL-mediated eNOS-dependent NO production [37].

The observations from this study of the BChE interactions can facilitate the development of more precise methods of analysis of such complexes. This methodology can be useful for research on the BChE interactions in the brain tissue, especially in the aspect of the pathogenesis and progression of AD. Human serum contains hundreds of proteins; some make strong interactions with other blood components, but most interactions are probably weak and transient. In all protein–protein or protein–ligand interaction studies, some false-positive results caused by nonspecific interactions appear. Therefore, good controls should be used when conducting experiments to obtain the correct conclusions. Using such a control, the resin without monoclonal antibodies, the specificity of the monoclonal antibody-bound proteins is visible in the tables in Figure 4. We also applied two different independent methods based on other mechanisms to pull down BChE–protein complexes. Based on the results from both approaches, we can more confidently build a model of such interactions. However, obtained results of BChE interactions should be treated as a model for further studies and confirmed by independent experiments using similar techniques and other antibodies by other research groups.

## 4. Materials and Methods

### 4.1. Isolation of BChE–Protein Complexes by Size-Exclusion Chromatography (SEC) of Serum Proteins. Fractionation of Human Serum Proteins by HPLC

The sample 100 µL of serum was applied on the TSK gel 3000 SWXL (300 × 7.8 mm, Tosoh, Tokyo, Japan) equilibrated with the 10 mM phosphate buffer (PB) pH 7.4; 8 g/L NaCl at a flow rate of 1 mL/min at RT. The column was connected to a Merck Hitachi LaChrom HPLC system equipped with the L-7420 UV-Vis detector (Merck Hitachi LaChrom, Tokyo, Japan). Elution of proteins was monitored at 280 nm. Thirteen fractions of 200 µL were collected every 12 s in separate tubes. Anonymized serum samples were obtained from a collection of the Central Clinical Laboratory of the Medical University of Gdansk.

### 4.2. Determination of BChE Activity in the Fractions by Ellman’s Assay

BChE activity of each fraction was determined spectrophotometrically by modified Ellman′s method using BTC (S-butyrylthiocholine iodide) as a substrate. The assay was performed in 96-well microtiter plates in a final reaction volume of 200 μL of 100 mM PB buffer (pH 7.4) with a final concentration of 0.5 mM DTNB and 5 mM BTC. The absorbance was monitored at 412 nm by repeated measurements at 1 min intervals for 10 min by a thermostated microplate reader spectrophotometer (Tecan Infinite M200 Pro, Tecan Group Ltd., Männedorf, Switzerland) at 25 °C. The activity of 10 µL of each fraction as absorbance at 412 nm was shown in red numbers above corresponding bars.

### 4.3. Analysis of Protein Complexes by Native Polyacrylamide Gel Electrophoresis 

The volume of 10 µL of each fraction was separated on precast polyacrylamide gels (Mini-PROTEAN 4–15% TGX Stain-Free Gels (Bio-Rad, Hercules, CA, USA) under native conditions. PAGE was performed in TB (50 mM Tris, 25 mM boric acid) buffer at 4 °C (100 V, 90 min). The gel was then stain-free activated for 45 s and imaged using the ChemiDoc Touch imaging system (Bio-Rad, Hercules, CA, USA) and ImageLab software (version 6.0) (Bio-Rad, Hercules, CA, USA).

### 4.4. SDS-PAGE and Western Blotting

Each sample, 10 µL of collected fractions, was mixed with 5 µL Laemmli sample buffer containing 5% β-mercaptoethanol (BME) and subjected to electrophoresis on Mini-PROTEAN 4–15% precast TGX Stain-Free gels (Bio-Rad, Hercules, CA, USA) and run until the sample front had passed through the gel, approx. 45 min at 200 V. The gel was then stain-free activated after SDS electrophoresis and imaged as described above. The activated gel was transferred to a PVDF membrane (Bio-Rad, Hercules, CA, USA) using a wet transfer system in transfer buffer (25 mM Tris, 192 mM glycine) for 90 min at 100 V. The PVDF membrane was blocked with gentle agitation in TBST buffer (0.1% Tween 20 and 150 mM NaCl in 10 mM Tris–HCL, pH 7.4) with 3% BSA. Primary monoclonal antibodies were mixed in TBST buffer with 3% BSA and incubated overnight at 4 °C. After incubation with primary antibodies, the blotting membrane was washed 3 × 10 min with TBST. For immunodetection, a goat anti-rabbit or anti-mouse IgG horseradish peroxidase conjugate (diluted 1:3000, Bio-Rad) and an enhanced chemiluminescence kit (Clarity Western ECL Substrate, BioRad, Hercules, CA, USA) were used.

### 4.5. Binding of BChE–Protein Complexes by Affinity Chromatography on Hupresin 

BChE–protein complexes from fractions 6, 8, and 10 were purified on Hupresin AC Sepharose (Chemforase, Mont-Saint-Aignan, France). Hupresin AC was equilibrated with 100 mM NaCl and 20 mM Tris-HCl pH 7.4. Fractions were loaded on the 50 µL resin in 1.5 mL tubes and washed 2 times only using 200 µL 100 mM NaCl, 20 mM Tris-HCl pH 7.4 buffer. BChE–protein complexes were eluted with 50 µL of 0.5 M trimethylammonium chloride (TMA Cl) in 100 mM NaCl, 20 mM Tris-HCl pH 7.4 buffer. Eluted proteins were dialyzed and concentrated to 20 µL in Amicon Ultra 10 K Centrifugal Filters (Merck, Darmstadt, Germany).

### 4.6. Mass Spectrometry of Proteins

The protein samples were analyzed by mass spectrometry as described earlier [47]. MS analysis was performed in the Laboratory of Mass Spectrometry (IBB PAS, Warsaw). Fifty microliters of 100 mM ammonium bicarbonate buffer was added to each protein sample, which was reduced with 5 mM TCEP for 30 min at 60 °C, blocked with 10 mM MMTS for 15 min at RT, and digested overnight, shaking with 10 ng/mL trypsin (CAT NO V5280, Promega, Madison, WI, USA) at 37 °C. Finally, to stop digestion, trifluoroacetic acid was added at a final concentration of 0.1%. The digest was centrifuged at 4 °C and 14,000× *g* for 30 min to pellet solids. The particle-free supernatant was analyzed by LC-MS/MS in the Laboratory of Mass Spectrometry (IBB PAS, Warsaw, Poland) using a nanoAcquity UPLC system (Waters) coupled to an Orbitrap QExative mass spectrometer (Thermo Fisher Scientific, Waltham, MA, USA). The mass spectrometer was operated in the data-dependent MS2 mode, and data were acquired in the m/z range of 300–2000. Peptides were separated by a 180 min linear gradient of 95% solution A (0.1% formic acid in water) to 35% solution B (acetonitrile and 0.1% formic acid). The measurement of each sample was preceded by three washing runs to avoid cross-contamination. The final MS washing run was searched for the presence of cross-contamination between samples. Data were searched with Mascot (MatrixScience, Boston, MA, USA) against two protein databases: UniProt human reference database and homemade protein database including isoforms of BChE and the following parameters: enzyme trypsin; peptide mass tolerance 20 ppm; fragment mass tolerance 0.6 Da; fixed modification methylthio C; variable modification oxidation M.

### 4.7. Isolation of BChE–Protein Complexes by Fractionation of Blood Serum Proteins Using Density Iodixanol Gradient Ultracentrifugation 

After removing chylomicrons, 9.6 mL of serum was mixed with 2.4 mL of OptiPrep (Merck, Darmstadt, Germany) (60% (w/v) solution of iodixanol in water), transferred to a centrifuge tube, and centrifuged for 6 h at 54,000 rpm and 16 °C. Fifteen fractions were collected from the top of the gradient.

### 4.8. Sudan Black Stained Agarose Gel Electrophoresis Profile of Separated Fractions 

A volume of 1 µL of each fraction was mixed with 2 µL Sudan black 0.5% in DMSO and 7 µL of water. Mixed samples were loaded on 1% agarose gel in TB buffer (TB buffer (pH = 8.7)—Tris 50 mM, Boric acid 25 mM) and separated for 90 min at 100 V and 4 °C. Ellman’s reaction was developed in 100 mM PB buffer (pH 7.4) with a final concentration of 0.5 mM DTNB and 5 mM BTC.

### 4.9. Isolation of the Protein Complexes by Immunoprecipitation and Mass Spectrometry

Isolation of native protein complexes from fractions was performed using immobilizing monoclonal antibodies on the agarose support according to the instruction of the Thermo Scientific Pierce Co-Immunoprecipitation (Co-IP) Kit (Thermo Fisher Scientific, Waltham, MA, USA). Briefly, 10 µg of mouse anti-BChE monoclonal antibody (3E8) was immobilized on the AminoLink Plus Coupling Resin. Then, 200 µL of the fraction was added to the spin column containing the antibody-coupled resin and incubated overnight at 4 °C. The resin was washed two times with 200 μL IP Lysis/Wash Buffer, and complexes were eluted three times using elution buffer. Eluted proteins were dialyzed and concentrated to 20 µL in Amicon Ultra 10 K Centrifugal Filters (Merck, Darmstadt, Germany). The protein samples were analyzed by mass spectrometry as described above.

## Data Availability

The data that support the findings of this study are available from the corresponding author (J.J.), upon request.
